# 2537. Population Pharmacokinetics of Isavuconazole in Pediatric Patients

**DOI:** 10.1093/ofid/ofad500.2154

**Published:** 2023-11-27

**Authors:** Amit Desai, Dolly A Parasrampuria, Laura Kovanda, Shamim Sinnar, Stacey Tannenbaum

**Affiliations:** Astellas Pharma Global Development, Northbrook, IL, Northbrook, Illinois; Astellas Pharma Global Development Inc., Northbrook, Illinois, USA, Northbrook, Illinois; Astellas Pharma Global Development Inc., Northbrook, Illinois, USA, Northbrook, Illinois; Astellas Pharma Global Development Inc., Northbrook, Illinois, USA, Northbrook, Illinois; Astellas Pharma Global Development Inc., Northbrook, Illinois, USA, Northbrook, Illinois

## Abstract

**Background:**

Isavuconazole (ISAV) is approved for the treatment of invasive aspergillosis (IA) and invasive mucormycosis (IM) in adults; however, there are limited data for its use in pediatric participants. The aim of this analysis is to develop an integrated population pharmacokinetic (PPK) model from two pediatric studies and to derive area under the curve values at steady state (AUCss) to confirm the recommended clinical dose of isavuconazonium sulfate (ISAVUSULF) in pediatric population.

**Methods:**

Previously, a PPK model was developed for the pediatric population based on data from NCT03241550 (Phase 1 Pediatric PK study) and from NCT01555866, a phase 1 adult study in healthy and mildly renally impaired participants. Adult data was added to stabilize the model due to limited sample size of PK in pediatric population. PK Data from NCT03816176, a recently completed phase 2 pediatric study, was added to the existing data and the model was updated. AUC_ss_ were derived from the PPK model for the pediatric participants. Derived exposures were evaluated to see if they fell within the established target exposure range of 60 mg·hr/L to 230 mg·hr/L, thus allowing for bridging of adult efficacy and safety data to pediatric population.

**Results:**

The best model based on updated data was a 3-compartment model with combined zero and first order absorption and linear elimination and inter-individual variability on clearance, volumes of distribution of peripheral compartments, and on inter-compartmental clearance. The model also included allometric scaling based on body weight to scale size-related changes in clearance and volume of distribution. No covariates (age, sex, liver function tests) were significant. The mean derived AUC_ss_ for pediatric participants, irrespective of route of administration and age group, were within the established exposure range of 60 mg·hr/L to 233 mg·hr/L (Figure 1).

**Figure:**
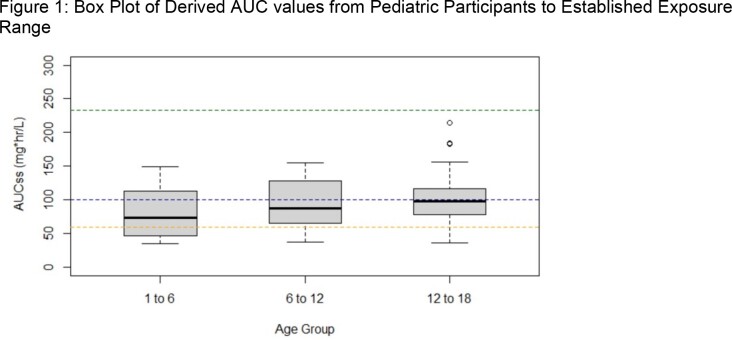

Box-and-whisker plots of drug exposure AUCss in pediatric age groups (1 to < 6, 6 to < 12, 12 to <18 years). Boxes represent the median and 25th and 75th percentiles, whiskers represent the range of maximum and minimum values within 1.5× the interquartile range, and outliers are shown as circles. Dashed blue line is the mean AUCss (100 mg·hr/L) from SECURE study. Dashed green line is the minimum (233 mg·hr/L) AUC24 value in a high dose adult study (1116 mg) with increased toxicity. Dashed orange line is the lowest targeted value (25th percentile, with AUCss of 60 mg·hr/L) based on exposures from SECURE study.

**Conclusion:**

The exposure data from pediatric studies allows bridging of the adult efficacy and safety data to pediatric patients. Based on the totality of the data, the proposed dosage regimen administered either as IV (1 to < 18 years) or PO (6 to < 18 years, with minimum weight of 12 kg) is:

- 10 mg/kg ISAVUSULF for patients weighing ≤ 37 kg or
- 372 mg ISAVUSULF for patients weighing > 37 kg.

**Disclosures:**

**Amit Desai, PhD**, Astellas Pharma Global Development, Inc.: Astellas Employee **Dolly A. Parasrampuria, PhD**, Astellas Pharma Global Development, Inc.: Stocks/Bonds|Johnson & Johnson: Stocks/Bonds **Laura Kovanda, PhD**, Astellas Pharma Global Development Inc.: Astellas Employee **Shamim Sinnar, MD, PhD**, Astellas Pharma Global Development, Inc.: Astellas Employee **Stacey Tannenbaum, PhD**, Astellas Pharma Global Development, Inc.: Former Employee|Metrum Research Group: Employee

